# Phosphorylation Modification of Wheat Lectin VER2 Is Associated with Vernalization-Induced *O*-GlcNAc Signaling and Intracellular Motility

**DOI:** 10.1371/journal.pone.0004854

**Published:** 2009-03-16

**Authors:** Lijing Xing, Juan Li, Yunyuan Xu, Zhihong Xu, Kang Chong

**Affiliations:** 1 Research Center for Molecular Developmental Biology, Key Laboratory of Photosynthesis and Environmental Molecular Physiology, Institute of Botany, The Chinese Academy of Sciences, Beijing, China; 2 National Centre for Plant Gene Research, Beijing, China; Cairo University, Egypt

## Abstract

**Background:**

*O*-linked β-N-acetylglucosamine (*O*-GlcNAc) modification of proteins mediates stress response and cellular motility in animal cells. The plant lectin concanavalin A can increase nuclear *O*-GlcNAc levels and decrease cytoplasmic *O*-GlcNAc levels in T lymphocytes. However, the functions of *O*-GlcNAc signaling in plants, as well as the relation between plant lectins and *O*-GlcNAc in response to environmental stimuli are largely undefined.

**Methodology/Principal Findings:**

We describe a jacalin-like lectin VER2 in wheat that shows N-acetylglucosamine and galactose specificity. Immunocytochemical localization showed VER2 expression induced predominantly at potential nuclear structures in shoot tips and young leaves and weakly in cytoplasm in response to vernalization. In contrast, under devernalization (continuous stimulation with a higher temperature after vernalization), VER2 signals appeared predominantly in cytoplasm. 2-D electrophoresis, together with western blot analysis, showed phosphorylation modification of VER2 under vernalization. Immunoblot assay with *O*-GlcNAc-specific antibody revealed that vernalization increased *O*-GlcNAc modification of proteins at the global level. An *O*-GlcNAc-modified protein co-immunoprecipitated with VER2 in vernalized wheat plants but not in devernalized materials. The dynamic of VER2 was observed in transgenic *Arabidopsis* overexpressing the VER2-GFP fusion protein. Overexpressed VER2 accelerated nuclear migration. Immunogold labeling and indirect immunofluoresence colocalization assay indicated that VER2-GFP was targeted to the secretory pathway.

**Conclusions/Significance:**

*O*-GlcNAc signaling is involved in the vernalization response in wheat, and phosphorylation is necessary for the lectin VER2 involving *O*-GlcNAc signaling during vernalization. Our findings open the way to studies of *O*-GlcNAc protein modification in response to environmental signals in plants.

## Introduction

Plant lectins are specific carbohydrate-binding proteins classified into various families. Jacalin-related lectins (JRL) are further subdivided into the galactose- and mannose-specific groups [1]. On the basis of their carbohydrate-binding characteristics, plant lectins are also divided into “classical” and “non-classical” lectins [2]. Classical lectins are usually abundant proteins with protein storage and plant defense properties in some plant tissues. In contrast, non-classical lectins induced by exogenous or endogenous stimuli are involved in specific protein-carbohydrate interactions and are suggested to play specific endogenous roles in plant tissues or cells [2].

Jacalin-related mannose-specific lectins have been reported in monocotyledonous plants. Orysata, a mannose-specific jacalin in rice, is a potent mitogen of T lymphocytes and is involved in stress defense-related protein-carbohydrate interactions in plants [3]. Recently, horcolin, a new jacalin-related lectin specific to mannose, from *Hordeum vulgare*, was proposed to perceive and transfer environmental stress signaling [4]. Despite the well-characterized carbohydrate-binding activity of plant lectins, cellular signaling and regulation mediated by the specific interaction of plant lectins with glycosylated proteins and glycoconjugates remains to be clarified. Nictaba, a tobacco lectin, is localized in the cytoplasm and nucleus, and the nuclear distribution of the protein is directed by a nuclear localization signal [5]. The constitutively overexpressed EGFP-Nictaba fusion protein showed lectin activity. High affinity of Nictaba for N-glycans suggested that it interacts with N-glycosylated nuclear proteins by binding to the nuclear envelope [6]. Although N-glycosylated cytoplasm and nuclear proteins possibly play important roles by interacting with lectins, nucleocytoplasmic proteins modified by *O*-linked β-*N*-acetylglucosamine (*O*-GlcNAc) are ubiquitous and of great significance in plant and animal cells [7]. *O*-GlcNacylation, similar to *O*-phosphorylation, is a dynamic post-translational modification at serine or threonine residues of nuclear and cytoplasmic proteins [8]. *O*-GlcNAc transferase (OGT) and neutral β-*N*-acetylglucosaminidase are responsible for the addition and removal of *O*-GlcNAc [9]. In animal cells, *O*-GlcNAc has been well characterized and shown to play functional roles in stress response [10], cell cycle progression [11] and cellular motility [12]. Nucleocytoplasmic proteins modified by *O*-GlcNAc include transcription factors [13], vesicular trafficking proteins [14], cytoskeletal proteins and nuclear pore proteins [9].

*O*-GlcNAcylation and *O*-phosphorylation of proteins have a reciprocal relation [15]. The two translational modifications also operate concomitantly on proteins [16]. Protein *O*-GlcNAc modification has been proposed to play a critical role in plant development [17]. Two OGT enzymes, SECRET AGENT (SEC) and SPINDLY (SPY), identified in *Arabidopsis*, show high similarity with animal OGT [18]–[20]. Nevertheless, the involvement of *O*-GlcNAc in the physiological process and cellular regulation is largely unknown in plants. The limitation of detection methods, to a great extent, has resulted in an impediment to the research on *O*-GlcNAc modification.

We previously reported that transcription of *VER2* is induced by vernalization and jasmonate acid in wheat and that a jacalin-like domain exists at the C terminus [21]. Vernalization refers to the process of prolonged cold inducing the promotion of flowering in plants. Significant progress has been made in understanding the molecular mechanisms of vernalization in the model plant *Arabidopsis* [22]–[25]. Winter wheat is one of the major food crops requiring vernalization for the development of flowering organs. The molecular mechanisms for cereals responding to vernalization differ from that for *Arabidopsis* [26], [27]. Methylation and acetylation modification of histones were shown to control epigenetic memory of winter in *Arabidopsis* [28]–[31]. Compared with the well-elucidated molecular and biochemical mechanisms of vernalization in *Arabidopsis*, those for winter wheat remain to be further elucidated.

In the current study, we characterized the lectin activity of VER2 and analyzed variation of protein *O*-GlcNAc modification at the global level in response to vernalization. The subcellular location and dynamics of the overexpressed VER2-GFP fusion protein was investigated in transgenic *Arabidopsis*. Our data suggest that *O*-GlcNAc glycosylation participates in the vernalization response in wheat, and phosphorylation of VER2 is necessary to mediate *O*-GlcNAc signaling and its compartmentalization induced by vernalization.

## Results

### Lectin activity analysis of VER2

**Figure 1:**
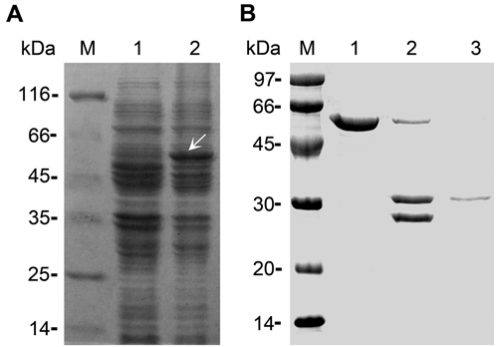
Purification of VER2 protein. (A) Induction of GST-VER2 recombinant protein in *E. coli* by use of IPTG. Lane 1, cell lysate before the addition of IPTG; Lane 2, cell lysate at 5 h after IPTG induction. The position of induced GST-VER2 is marked by an arrow. Lane M, molecular weight markers. (B) Purification of recombinant VER2 protein. Lane 1, the purified GST-VER2 fusion protein; Lane 2, thrombin digestion of GST-VER2 to GST and VER2; Lane 3, the purified VER2. Lane M, molecular weight markers. The gels were stained with coomassie brilliant blue.

To confirm the lectin activity of VER2, 1 mM isopropyl-β-D-thiogalactoside (IPTG) was used to induce the expression of GST-VER2 fusion protein in *Escherichia coli* BL21. A protein band matching the expected size of GST-VER2, at approximately 58 kDa, was visible at 3 h after incubation (Figure 1A, lane 2). The fusion protein was affinity purified and the eluted VER2-GST fusion protein was cleaved with thrombin to release VER2 protein (Figure 1B, lane 2). The purified VER2 was able to agglutinate rabbit erythrocytes at a minimum concentration of 1.5 µg/ml. On the basis of the jacalin-like domain at the C terminus of VER2, carbohydrate-binding specificity was detected with mannose, galactose, N-acetyl-D-glucosamine, glucose, sucrose, fructose, maltose and albumin egg. These sugars were tested in hapten inhibition assay to analyze their inhibitory effect on agglutination of 2% rabbit erythrocyte suspension. As shown in Table 1, the agglutination activity of VER2 was readily inhibited by N-acetyl-D-glucosamine and galactose, with the lowest inhibition concentration of 3.1 mM and 6.25 mM, respectively, and VER2 interacts more specifically with N-acetyl-D-glucosamine. In contrast, mannose could not inhibit the agglutination. Fructose, glucose and maltose were less sensitive. Sucrose was 8 times less sensitive than galactose, which suggests that the carbohydrate binding sites of VER2 are more adaptive to monosaccharide than disaccharide. The glycoprotein albumin egg also had an inhibitory effect because of the N-acetyl-D-galactose ligands on its molecular structure.

**Table 1:** Comparison of the carbohydrate-binding specificities of VER2.

Sugar/Glycoprotein	MIC
Galactose	6.25 mM
Glucose	25 mM
N-Acetyl-D-glucosamine	3.1 mM
Mannose	>100 mM
Sucrose	50 mM
Fructose	12.5 mM
Maltose	25 mM
Albumin Egg	6.25 µg/ml

Solutions containing purified VER2 and simple sugars were preincubated for 1 h at room temperature, then rabbit erythrocytes suspension was added, respectively, and the agglutination was evaluated after 1 h. MIC: The lowest concentration of sugars or glycoproteins at which the inhibition of the agglutination was visible.

### Immunocytochemical localization of VER2 in response to vernalization in wheat

**Figure 2:**
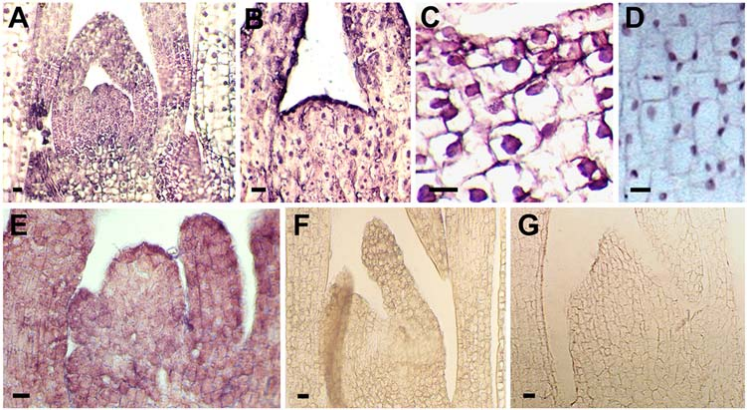
Immunocytochemical localization of VER2, showing labeling signals in shoot apex and young leaves. Sections were probed with anti-VER2 antibody followed by a goat anti-rabbit alkaline phosphatase (AP)-conjugated secondary antibody. (A) Plants were vernalized for 21 days. VER2 is predominantly targeted to potential nuclear structures. Weaker labeling was detected in cytoplasm. (B) An enlarged image showing labeling signals in shoot apical meristem. (C) An enlarged image showing labeling signals in young leaves. (D) Showing hematoxylin stained nuclei of young leaves. (E) Plants were first vernalized for 21 days, then devernalized. Signals were dispersed in the cytoplasm. (F) No immunocytochemical signal detected in nonvernalized plants. (G) Negative control performed by omitting the first antibody. Bars, 20 µm.

Previous *in situ* hybridization results showed that vernalization induces the mRNA expression of *VER2* [21]. Here, protein immunocytochemistry analysis was used to determine the spatial and temporal expression patterns of VER2 in response to vernalization and devernalization. Devernalization treatment is a valuable control system of vernalization at the morphological or physiological level. The specificity of the antibody prepared with purified VER2 was detected by western blot analysis before immunocytochemical labelling. The anti-VER2 antibody specifically recognized a protein band at the expected molecular weight for VER2 (Figure S1) in wheat plants vernalized for 3 weeks. Furthermore, the labelling signal from the anti-VER2 antibody was consistent with the labelling results for antibody prepared with a synthesized polypeptide of VER2 [32]. Vernalized, devernalized and non-vernalized wheat plants were used for immunolabeling. Labeling signals were detected in shoot apical meristem and young leaves of plumules vernalized for 3 weeks (Figure 2A to C). Compared with nuclei indicated by hematoxylin staining in young leaves (Figure 2D), VER2 protein was shown to target predominantly to potential nuclear structures. In devernalized plants, immunolabeling signals were detected only in cytoplasm (Figure 2E), with no signal detected in nonvernalized plants (Figure 2F) or in the negative control (Figure 2G) under the same conditions. As well, immunolabeling signals were not detected in plants vernalized for 1 and 2 weeks (data not shown).

### Vernalization induces phosphorylation modification of VER2 in wheat

**Figure 3:**
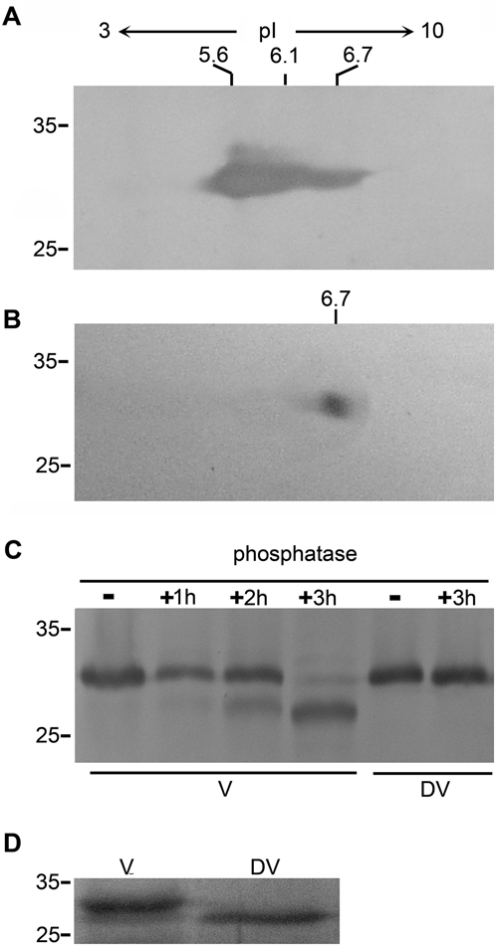
Immunoblot analysis of VER2 separated on 2-D electrophoresis and dephosphorylation of VER2 by phosphatase in vernalized and devernalized wheat plants. (A) VER2 was separated into 3 main spots with pI = 5.6, 6.1 and 6.7, respectively, in vernalized wheat plants. (B) VER2 was detected as one spot with pI = 6.7 in devernalized wheat plants. (C) VER2 was immunoprecipitated and then treated with protein phosphatase for the time shown in vernalized and devernalized plants. (D) Immunoblot analysis showing the difference of migration distance of VER2 in vernalized and devernalized materials. The samples were separated by electrophoresis for enough time until the marker band with the molecular weight of 25 kDa shifted to the forefront of the 8 cm gel.

The nucleocytoplasmic exchange of lectin plays a role in response to osmotic stress in yeast cells [33]. Amino acid sequence analysis indicated that VER2 does not contain a signal sequence for subcellular targeting. The different subcellular localization patterns of VER2 in response to vernalization and devernalization indicated that post-translational modification of VER2 might be involved in regulating its intracellular targeting. To address this possibility, total proteins from vernalized and devernalized materials were analyzed by 2-D gel electrophoresis and immunoblotted with anti-VER2 antibody. Continuous signal spots were apparent in wheat samples vernalized for 3 weeks (Figure 3A). Nevertheless, only a single spot was detected in devernalized plants (Figure 3B). Compared with the predicted isoelectric point (pI) of 6.6 (http://us.expasy.org/tools/pi_tool.html) of VER2, the signal detected from vernalized plants (Figure 3A) showed an acidic shift. In the devernalized sample, the immunoblotting signal was at the approximate expected pI of VER2, which suggested that vernalization could induce post-translational phosphorylation modification of VER2.

To further confirm the phosphorylation modification of VER2 in vernalized wheat plants, VER2 in vernalized and devernalized materials was immunoprecipitated and treated with protein phosphatase for 1, 2 and 3 h, then analyzed by immunoblotting. VER2 in vernalized plants was detected as two bands. Such mobility shift indicated that the fast migrating band in vernalized samples is the dephosphorylation form of VER2. The level of the dephosphorylated form of VER2 increased with prolonged treatment time. No band shift of VER2 occurred in the devernalized sample after 3 h of phosphatase treatment (Figure 3C). Because of lack of difference in shift distance of VER2 before phosphatase treatment in vernalized and devernalized materials due to the shorter migration distance, samples for SDS-PAGE were separated for more time before immunoblotting analysis. The following immunoblot result showed that VER2 in devernalized material migrated faster than that in vernalized material when the marker band of 25 kDa was allowed to move to the forefront of the 8-cm gel (Figure 3D). Thus, the difference in migration distance of VER2 between vernalized and devernalized wheat plants resulted from the post-translational phosphorylation modification of VER2 induced by vernalization.

### Vernalization induces an increase of *O*-GlcNAc modification of proteins at the global level and the binding of VER2 to *O*-GlcNAc-modified protein(s) in wheat

**Figure 4:**
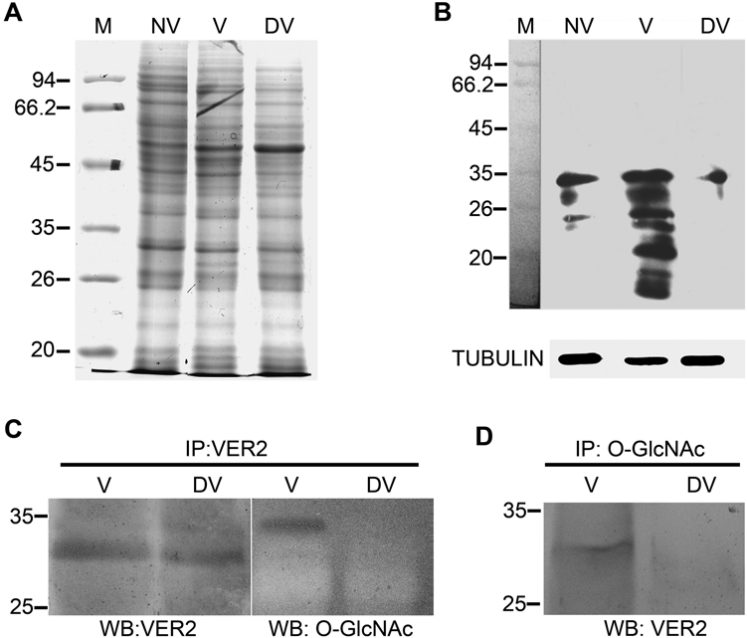
Detection of *O*-GlcNAc-modified proteins and their association with VER2 in vernalized and devernalized wheat plants. (A) SDS-PAGE results stained with coomassie blue. (B) Immunoblot analysis of *O*-GlcNAc-modified proteins in nonvernalized, vernalized and devernalized plants with *O*-GlcNAc site-specific antibody CTD110.6. Tubulin was immunoblotted as a loading control of total proteins with anti-tubulin polyclonal antibody. (C) Proteins from vernalized and devernalized wheat plants were immunoprecipitated with anti-VER2 antibody and detected with anti-VER2 and CTD110.6 antibodies, respectively. An *O*-GlcNAc modified protein with a molecular weight of about 35 kD was identified in VER2 immunoprecipitates from vernalized materials; no blotting signals for *O*-GlcNAcylated proteins were detected in devernalized materials. (D) VER2 was identified in *O*-GlcNAc immunoprecipitates from vernalized plants but not from devernalized plants. M, molecular weight markers; NV, nonvernalized; V, vernalized; DV, devernalized. IP, immunoprecipitate; WB, western blotting.

To test whether the *O*-GlcNAc modification of proteins is affected by vernalization, which could be regarded as one kind of special environmental stress, we detected the variation of *O*-GlcNAc modified proteins at the global level in response to vernalization and explored the possibility of VER2 binding with *O*-GlcNAcylated proteins because of the carbohydrate specificity of VER2 to N-acetylglucosamine. We detected *O*-GlcNAc-modified proteins in nonvernalized, vernalized and devernalized wheat plants using the monoclonal antibody CTD110.6 originally produced to specifically recognize Ser- or Thr-*O*-GlcNAc-modified proteins in animal cells [34]. Western blot analysis showed that vernalization increased the global level of *O*-GlcNAc-modified proteins as compared with nonvernalization; furthermore, devernalization decreased the level of *O*-GlcNAc-modified total proteins, with only a few proteins detected (Figure 4A, B). We then immunoprecipitated VER2 from vernalized and devernalized extracts of wheat plumules using the anti-VER2 antibody. The antibody CTD110.6 was used to detect *O*-GlcNAc-modified proteins in immunoprecipitates. A protein band of approximately 35 kDa was detected in vernalized plants. In contrast, no signal was detected in devernalized materials (Figure 4C). Neither vernalized nor devernalized plants showed *O*-GlcNAc-modified signals corresponding to VER2. Figure 4D shows that VER2 immunoprecipitated with *O*-GlcNAc-modified proteins in vernalized but not devernalized plants.

### Subcellular location and intracellular motility of VER2-GFP in transgenic *Arabidopsis*

**Figure 5:**
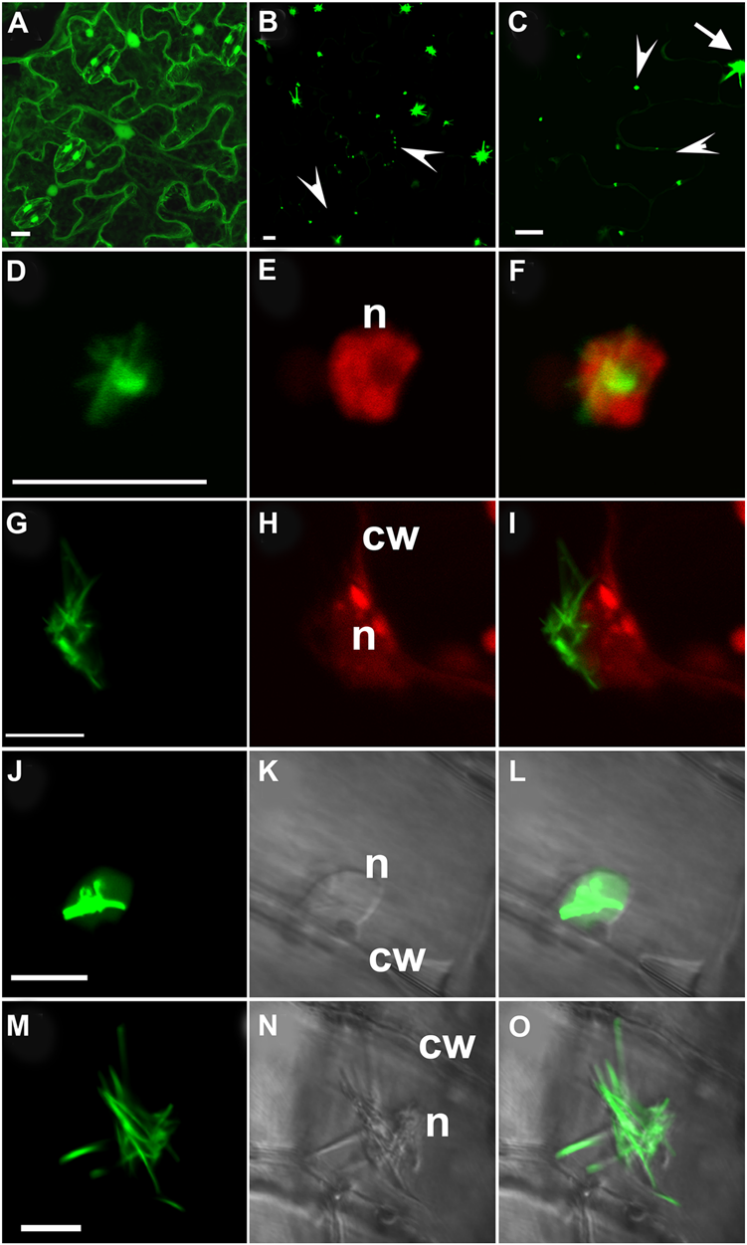
Confocal images of VER2-GFP fusion protein and GFP overexpressed in leaf epidermis and veins in *Arabidopsis*. At least 6 independent transgenic plants expressing VER2-GFP and GFP were analyzed. GFP fluorescence is shown in the green channel. Propidium Iodide (PI) fluorescence is shown in the red channel. (A) Localization of GFP alone in epidermal cells. (B) Localization of VER2-GFP fusion protein in epidermal cells. The arrowheads point to punctate distribution of VER2-GFP. (C) An enlarged image of VER2-GFP fusion protein in epidermal cells. The arrowheads point to punctate distribution of VER2-GFP. Nuclear and perinuclear distributed VER2-GFP was labeled with an arrow. (D), (E) and (F) Enlarged images of fluorescence of nuclear and perinuclear distributed VER2-GFP in leaf epidermal cells. The nucleus in (D) was stained with PI and shown in (E). The emerged image of (D) and (E) was shown in (F). (G), (H) and (I) Enlarged images of fluorescence of perinuclear distributed VER2-GFP in leaf epidermal cells. The nucleus in (G) was stained with PI and shown in (H). The emerged image of (G) and (H) was shown in (I). (J), (K) and (I) Enlarged images of fluorescence of nuclear and perinuclear distributed VER2-GFP in vein cells. Transmitted light image of (J) was shown in (K). The emerged image of (J) and (K) was shown in (L). (M), (N) and (O) Enlarged images of fluorescence of perinuclear distributed VER2-GFP in vein cells. Transmitted light image of (M) was shown in (N). The emerged image of (M) and (N) was shown in (O). n, nucleus; cw, cell wall. Bars, 10 µm.

We generated *35S::VER2-GFP* and *35S::GFP* transgenic *Arabidopsis* plants as a model system to determine the precise subcellular location pattern and dynamics of VER2 and further understand its function. GFP alone was observed in the cytoplasm and nucleus of leaf epidermal cells in *35S::GFP* transgenic plants (Figure 5D). However, VER2-GFP fluorescence showed aggregates with tubular extension, as well as punctate signals in cytoplasm (Figure 5B, C). To further confirm the nuclear targeting of VER2 as shown in Figure 2, *Arabidopsis* epidermis was stained with propidium iodide (PI) to indicate the position of nuclei. VER2-GFP was localized in the nucleus as well as perinuclear region in tubular structures (Figure 5D to F), or only at the perinuclear region in some epidermal cells (Figure 5G to I). Vein cells were arranged in a regular pattern and could be captured in clear bright-field images to confirm nuclear and perinuclear tubular distribution of VER2-GFP (Figure 5J to O).

**Figure 6:**
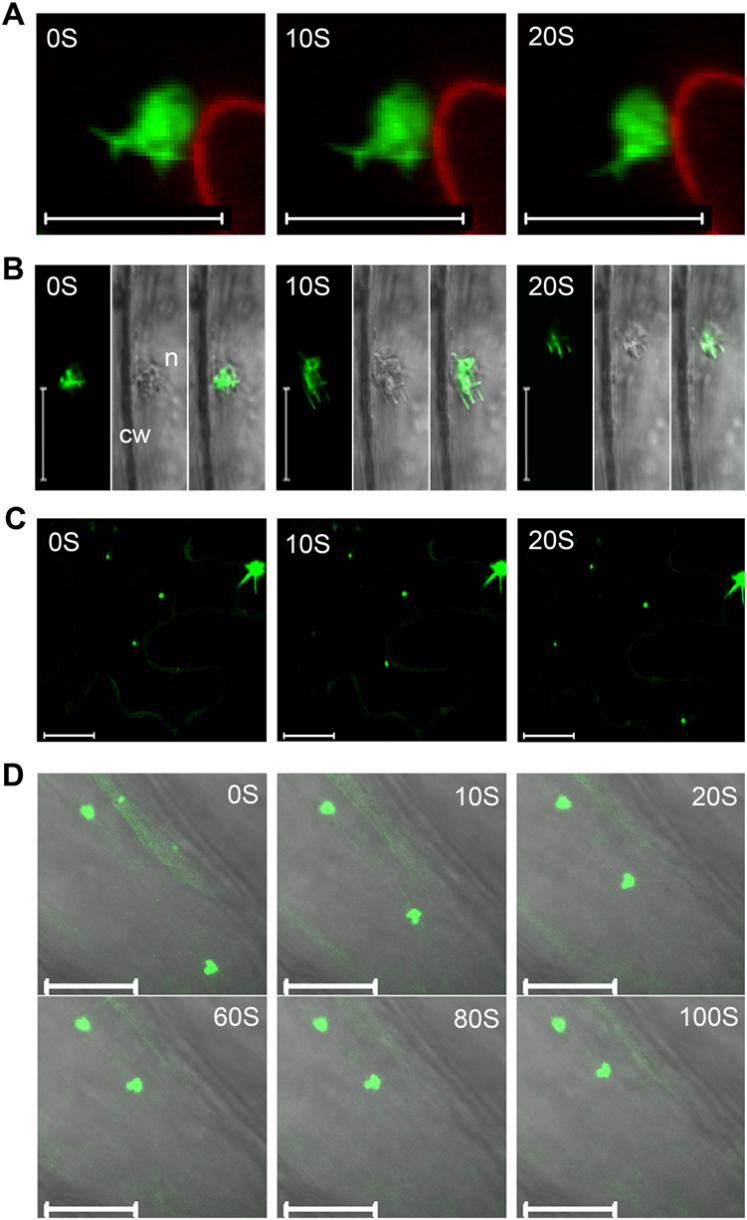
Motility of VER2-GFP fusion protein in *Arabidopsis* leaf epidemis and veins. (A) VER2-GFP involved nuclear movement in epidermal cells. PI-stained cell wall was shown in the red channel. (B) VER2-GFP involved nuclear movement in vein cells. (C) Movement of punctate-located VER2-GFP in leaf epidermal cells. (D) Temporal dynamics of VER2-GFP targeted to punctuate structures in vein cells, showing merged images of fluorescence and corresponding transmitted images. n, nuclei; cw, cell wall. Bars, 20 µm.

**Figure 7:**
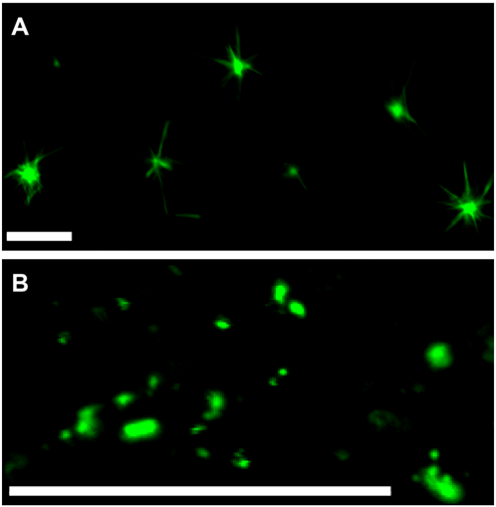
Perinuclear-distributed VER2-GFP is associated with microtubules in transgenic *Arabidopsis*. (A) Epidermal cells treated with DMSO (1%) for 30 min. (B) Epidermal cells treated with PPM (5 µM) for 30 min. Bars, 20 µm.

In epidermal cells of young leaves of transgenic *Arabidopsis* overexpressing VER2-GFP, VER2-carrying nuclear and perinuclear structures were observed to change direction randomly and move within the cell (Figure 6A). The proportion of observed cells with nuclear motility is about 10%. In vein cells, nuclei showed axial migration with a velocity of approximately 30 µm/min (Figure 6B), which is much faster than nuclear movement in *Arabidopsis* root hairs (<10 µm/min) [35]. However, nuclear migration in *35S::GFP* transgenic *Arabidopsis* plants could not be observed under the same conditions. Therefore, VER2-GFP could alter the motility and position of nuclei in transgenic plants. Overexpression of VER2 was suggested to facilitate nuclear motility. Punctate-targeted VER2-GFP was also observed with a mobile pattern in leaf epidermis and veins (Figure 6C,D), and mobile punctate signals could fuse together. Nuclei are reported to move along microtubules [36]. To examine whether perinuclear-distributed VER2-GFP is associated with cellular microtubules or moves passively with the nucleus, we treated *Arabidopsis* epidermis with the cytoskeleton-destabilizing agent propyzamid (5 µM). The propyzamid was dissolved in DMSO and diluted to working concentration to incubate *Arabidopsis* leaves. As a control, DMSO treatment alone did not alter the location pattern of VER2-GFP (Figure 7A), but treatment with propyzamid destroyed the perinuclear tubular extension of VER2-GFP (Figure 7B). Thus, VER2 overexpressed at the perinuclear region is associated with microtubules.

### VER2-GFP is targeted to the secretory pathway but not as a sorting receptor

**Figure 8:**
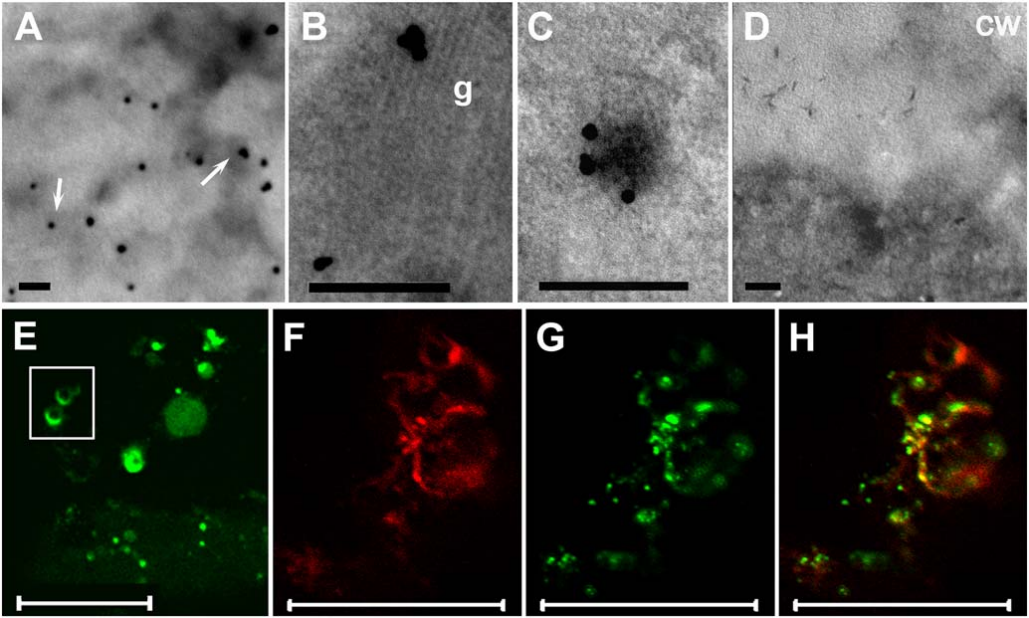
Immunogold labeling of VER2-GFP and colocalization of VER2-GFP with VSR_At_ in *Arabidopsis* leaves. (A)–(D) Immunogold labeling of VER2-GFP. Sections were incubated with anti-GFP antibody (A to C) or preimmune serum (D) followed by gold-conjugated secondary antibody. Gold particles accumulate on ER (A) Golgi apparatus (B) and PVC (C). No specific labeling was found in the control image (D). ER is indicated with arrows. Bars, 0.2 µm. (E) VER2-GFP fusion signals with ring structures. (F)–(H) Colocalization of VER2-GFP with VSR_At_. The anti-VSR-marked prevacuolar organelles are shown in red channel. Endogenous VSR was detected by indirect immunofluorescence labeling. Fixed leaves were stained with anti-VSR antibody followed by a TRITC-labeled anti-rabbit antibody (F). GFP signals indicated punctate distribution of VER2-GFP in green channel observed from the fixed leaves (G). Merged image of (F) and (G) is shown as (H). g, Golgi; cw, cell wall. Bars, 20 µm.

To identify organelles responsible for the mobile punctate distribution pattern of VER2-GFP in transgenic *Arabidopsis*, immunogold labeling was carried out using anti-GFP antibody and did not reveal a vacuolar distribution pattern of VER2-GFP as was reported for other lectins [37]. Gold particles located on the endoplasmic reticulum (ER) and Golgi apparatus and on the surface of uncoated vesicles (Figure 8A to D). This location pattern, combined with the ring punctate signals of VER2-GFP fluorescence (Figure 8E), suggests that VER2 targets to prevacuolar compartments (PVCs) reported in *Arabidopsis* [38], [39]. Vacuolar sorting receptor (VSR) is a marker protein of PVC involved in vacuolar sorting [40]. Confocal immunofluorescence microscopy revealed that VER2-GFP colocalized in part with endogenous VSR_At_ immunolabeled with anti-VSR antibody in transgenic *Arabidopsis* expressing VER2-GFP (Figure 8F to H). Thus, punctate labeling of VER2-GFP indicated the ER, Golgi and PVCs, which are the components of the vacuolar trafficking pathway.

The distribution of VER2 in the secretory pathway raises the possibility that VER2 acts as a sorting receptor of glycoproteins. The general domain organization of carbohydrate sorting receptors contains an exoplasmic domain with one or multiple carbohydrate recognition domains, a single transmembrane domain, and a cytoplasmic domain with sorting signals [41]. Nevertheless, unlike other known sorting receptors, such as ERGIC-53 and VSR, which have a typical transmembrane domain, VER2 did not display any of the main features of sorting receptors (http://www.cbs.dtu.dk/services/TMHMM/, http://mobyle.pasteur.fr/cgi-bin/MobylePortal/portal.py?form=toppred, and http://www.cbs.dtu.dk/services/TargetP/). VER2 is therefore unlikely a sorting receptor of *O*-GlcNAcylated proteins in the secretory pathway.

### The effects of site-directed mutagenesis of phosphorylation sites on the compartmentalization of VER2 in *Arabidopsis*

**Figure 9:**
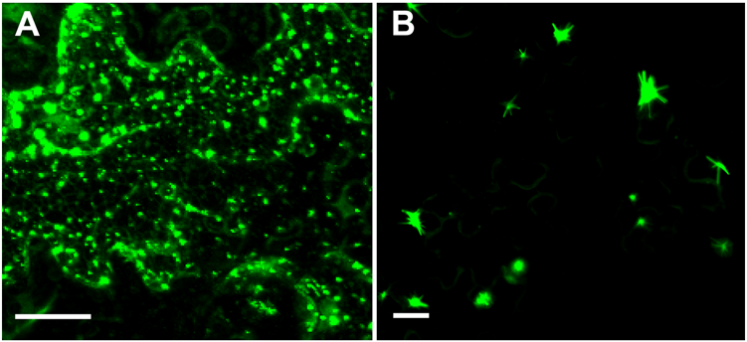
Location patterns of VER2-S33G and VER2-T209A mutations fused to GFP. (A) VER2-S33G mutation. (B) VER2-T209A mutation. Bars, 20 µm.

The amino acid sequence analysis of VER2 showed predicted serine and threonine phosphorylation sites and Yin-Yang sites, which have potential reciprocal dynamic phosphorylation and *O*-GlcNAc modification possibility (http://www.cbs.dtu.dk/services/NetPhos/) (http://www.cbs.dtu.dk/services/YinOYang/). The serine at site 33 and threonine at site 209 of the amino acid sequence are predicted Yin-Yang sites. Phosphorylation on these two sites is important for protein functioning. We generated transgenic *Arabidopsis* overexpressing mutated VER2 of S33G and T209A to observe the compartmentalization of fluorescence of GFP. The location pattern of mutant VER2-S33G is different from that of VER2. VER2S33G was predominantly targeted to punctate structures, and very weak location signaling could be seen in nuclei and cytoplasm (Figure 9A). Transgenic *Arabidopsis* plants expressing VER2-T209A showed no obvious differences in GFP fluorescence distribution (Figure 9B).

## Discussion

### VER2 is a JRL responding to vernalization in wheat

Plants are well known to contain lectins with carbohydrate binding and agglutination activity. JRL proteins are divided into galactose- and mannose-specific subgroups. We show here that VER2 is a JRL protein with N-acetylglucosamine and galactose specificity. Peumans *et al.* [1] reported that a mannose-specific jacalin, Calsepa, was localized in the cytoplasm, whereas a galactose-specific jacalin was shown to locate in small storage vacuoles. Nevertheless, another mannose-specific jacalin, MornigaM, with no transit peptides, showed considerable presence in the nucleus as well as cytoplasm. The nuclear location mechanism for MornigaM is not clear [37]. Nictaba has a typical nuclear localization signal corresponding to its nuclear location [5]. Nevertheless, the amino acid sequence of VER2 does not indicate any classical nuclear localization signals or signal peptides for subcellular targeting. However, VER2 was targeted to the nucleus, perinuclear region and cytoplasmic mobile punctate structures (Figures 2,5,6). These subcellular location patterns differed greatly from that of other reported lectins. Amino acid sequence analysis of VER2 showed a dirigent domain in the N terminus and a jacalin-like domain in the C terminus. We previously described a mannose-specific jacalin OsJAC1 with the same two domains as VER2 in rice [42], overexpressed OsJAC1-GFP showed the same location pattern as GFP alone in *Arabidopsis* (unpublished data). The N-terminal dirigent domain of plant lectins was suggested to play defensive or stress-related roles [43]. VER2 could act as a nonclassical lectin to respond to vernalization in wheat.

### Phosphorylation is necessary for VER2 functioning

Phosphorylation and dephosphorylation can direct cytoplasmic or nuclear localization of proteins in response to environmental stress and the nutrient state [33], [44], [45]. Our data clearly indicate that phosphorylation of VER2 is induced by vernalization. The phosphorylation modification of VER2 corresponds to its nuclear/perinuclear targeting and binding to *O*-GlcNAc-modified proteins. Removal of the phosphorylation state of VER2 eliminated the nuclear location and the binding of VER2 to *O*-GlcNAcylated protein. In transgenic *Arabidopsis*, site mutation of S33G disturbed phosphorylation modification of VER2 and changed its compartmentalization. We suggest that phosphorylated VER2 is involved in the vernalization response and that phosphorylation at S33 is necessary for VER2 functioning.

*O*-GlcNAc modification is an abundant and dynamic process, as is phosphorylation, for numerous nucleocytoplasmic proteins [46]. Cellular *O*-GlcNAc levels respond to environmental and nutrient stress in animal cells, and increasing global levels of *O*-GlcNAc-modified proteins increases stress tolerance and modulate the responsiveness of cells to extracellular signaling [47]. *O*-GlcNAc signaling and *O*-GlcNAcylated proteins in response to environmental cues are poorly understood in plants. Although SPY, the negative regulator of the gibberellic acid (GA) signaling pathway, has been identified in *Arabidopsis* as a homologue of *O*-GlcNAc transferase, *O*-GlcNAc-modified proteins involved in GA signal transduction have not been determined. In our study, vernalization as a special environmental stress increased protein *O*-GlcNAcylation at the global level in immunoblot assay with *O*-GlcNAc-specific antibody. Early studies determined the influence of carbohydrates on vernalization. Only exogenous sugar application could optimize vernalization of excised meristems. Glucose promotes and mannose inhibits vernalization [48]. Glucose may modulate vernalization through elevating cellular *O*-GlcNAc level. Our data indicate that vernalization needs the participation of *O*-GlcNAc-dependent intracellular signaling that may act as a novel regulatory signaling component. The relationship between *O*-GlcNAc modified proteins and plant lectins is largely undefined at present. Phosphorylated HSP-70, a nuclear/cytoplasmic GlcNAc-specific lectin from rat liver, was speculated to be involved in the shuttling of *O*-GlcNAcylation-modified proteins between cytoplasm and nucleus [16]. *O*-GlcNAc modification of HSP70 might mediate cellular protection by its binding to target proteins [49]. We speculate that phosphorylated VER2 may bind to O-GlcNAc modified protein(s) and regulate their stability and/or intracellular location during the vernalization process in winter wheat. The identification and functional analysis of *O*-GlcNAc-modified proteins induced by vernalization will be helpful to elucidate the mechanism of *O*-GlcNAc signaling in response to vernalization in plants, and additional studies of the relationship between VER2 regulation and *O*-GlcNAc modified proteins is of great importance.

### Overexpressed VER2 modulates nuclear movement in *Arabidopsis*

Nuclear migration is critical for proper developmental processes in eukaryotes and depends on microtubules and actin filaments [36], [50]. We have examined the nuclear movement and present evidence that overexpressed VER2 in transgenic *Arabidopsis* accelerated nuclear migration. Pre-mitotic nuclear migration is reported to occur in the G1 phase and the nuclear positioning indicates the potential division site [51]. During the post-mitotic process, nuclear migration includes the movement of nuclei toward daughter cells and the center position of interphase cells. Kinesin is involved in unidirectional nuclear motility and positioning [52], [53]. The migration of interphase nuclei in fungi has been described to be intermittent and related with other cell cycle events [54]. Here, the nuclear motility we observed is possibly pre-mitotic or positioning in cells of post-mitotic stage. The previously published data showed that the heading and maturation time was delayed and tissue degeneration was observed at the top of spikes in antisense transgenic wheat plants [21]. In the present study, we compared the expression of VER2 protein in roots, stems, leaves, and panicles at different development stages by western blotting. Before heading, VER2 was detected in leaves and panicles of wheat but not roots and stems. The protein level in panicles was higher than that in leaves. After heading, VER2 was expressed only in panicles. The immunoblotting signal of tubulin was used as a loading control of total proteins (Figure S2). The specific accumulation of VER2 protein during heading indicates that VER2 is required for panicle development in wheat. The rate of nuclear migration functions as a influencing factor of cell cycle duration [55]. Our results provide some indication that VER2 possibly participates in spike development via regulating nuclear motility-associated mitotic process during heading stage. Rice lectin Orysata exhibits mitogenetic activity towards T lymphocytes [3]. Mechanisms for plant lectins presenting mitotic activity need to be further elucidated.

### VER2 involved in intracellular motility may mediate *O*-GlcNAc signaling

In mammalian cells, lectins mediate glycoprotein transport along the secretory pathway. The transmembrane mannose-binding lectin ERGIC-53 homologous to leguminous acts as a receptor for sorting glycoproteins in the secretory pathway [56], [57]. The lectin domain of ERGIC-53 is responsible for sorting and transporting glycoproteins from the ER to Golgi apparatus [58]. The biochemical and cell biological mechanisms of lectins in the intracellular secretory pathway in plants are still unknown. N-linked and *O*-linked carbohydrates act as targeting signals for glycoprotein sorting by lectins from the Golgi apparatus to plasma membrane [59]. Glycoproteins with galactose, N-acetylglucosamine and N-acetylgalactosamine oligosaccharide chains were detected in the Golgi apparatus and plasma membrane [60]. In plant cells, cargo proteins are sorted to vacuoles or another intracellular trafficking pathway in the *trans*-Golgi network [61]. Proteins destined for vacuoles require sorting signals in polypeptides. N-terminal propeptide (NTPP) and C-terminal propeptide (CTPP) are vacuolar sorting determinants in plants [62]. The amino acid sequence of NPIRL is the conserved motif for vacuolar sorting of sporamin [63] and barley aleurain [64]. The CTPP of a barley lectin is necessary for its vacuolar sorting [65]. VSR1, an intermediate compartment for vacuolar sorting of cargo proteins, interacts with the NTPP but not CTPP of cargo proteins [66]. Amino acid sequence analysis of VER2 does not show a signal peptide, NTPP or CTPP. Therefore, the mobile transport clusters of VER2-GFP do not denote the transport of VER2 itself as a vacuolar storage component. By using dynamic fluorescence and indirect immunofluorescence co-localization analysis, we demonstrate that VER2 is associated with vacuolar trafficking. Although vernalization induced *O*-GlcNAc modification of many proteins, no more proteins were detected binding with VER2. The result also supports that VER2 does not act as a sorting receptor in the secretory pathway.

Vesicle trafficking proteins could be modified with *O*-GlcNAc in animal cells [7], [67]. In neutrophils, *O*-GlcNAcylation induced by GlcNH2 can increase cellular motility [12] and regulate activities of intermediates of Rac and PI3K involved in cellular migration [68]. In plant cells, the relation between *O*-GlcNAc and intracellular motility is largely unknown. We propose that VER2 binds with specific *O*-GlcNAc-modified proteins in the secretory pathway and mediates intracellular motility modulated by *O*-GlcNAc signals. Our findings provide novel insights into lectin function in the vernalization response and motility-associated signaling in plants. Further studies are required to define more precisely the role of VER2 in *O*-GlcNAc signaling induced by vernalization.

## Materials and Methods

### Plant materials, growth conditions, and devernalization treatment

Seeds of winter wheat (*Triticum aestivum* L.) were surface sterilized in 2% NaClO for 20 min, then rinsed overnight with flowing water. For all treatments, seeds were cultured under darkness. For vernalization treatment, seeds were sown on moist filter paper and grown at 4°C for 21 days. Nonvernalized control seeds were grown at 25°C for 3 days. Seedlings vernalized for 21 days were subsequently transferred to an incubator at 35°C for 5 days, defined as devernalization.

### Purification of GST-VER2 recombinant protein

Two primers were designed (5′-TAAGAATTCATGGCCAAATTCCAGATTAC-3′ and 5′-AATCTCGAGGACCGTGTAAACACCAAATG-3′) to amplify *VER2* from cDNA. The PCR product was excised with *Eco*RI and *Xho*I, then purified and cloned into the corresponding sites of the pGEX-4T-3 vector. The construct was verified by DNA sequencing. The GST-VER2 fusion protein was purified according to the manufacturer's instructions (Amersham Biosciences).

### Removal of GST tag from the recombinant protein and purification of VER2

To cleave the GST tag, the fusion protein was cleaved with thrombin at room temperature for 16 h according to the manufacturer's recommendations (Amersham Biosciences). GST was removed by column purification on glutathione sepharose 4B beads. Purified VER2 in the flow-through was collected.

For preparation of anti-VER2 antibody, purified VER2 was injected into rabbits to produce polyclonal antibody. The anti-VER2 antibody was used for western blot analysis.

### Hemagglutination and Hapten carbohydrate-binding specificity of VER2

Agglutination assays were carried out in 96-U-well plates in a final volume of 40 µL containing 20 µL of purified VER2 serially diluted in two-fold increments and 20 µl of a 2% suspension of rabbit erythrocytes, according to a two-fold serial dilution procedure [69]. Agglutination was assessed visually after 1 h at room temperature.

Hapten inhibition of the agglutination was determined as follows. A total of 20 µL of sugars or glycoprotein, including galactose, glucose, N-acetyl-D-glucosamine, mannose, sucrose, fructose, maltose, and albumin egg, was serially diluted with a starting concentration of 200 mM and tested for carbohydrate-binding specificity. Solutions containing 10 µl of purified VER2 and 10 µl sugars or glycoproteins were preincubated for 1 h at room temperature, then 20 µl of a 2% rabbit erythrocyte suspension was added, and the agglutination was evaluated after 1 h.

### Production and purification of peptide-specific antibody

A peptide of 12 amino acids (KRRTTDSRGGGN, amino acids 205 to 214) predicted to be highly specific to VER2 was synthesized [32]. The peptide was linked to BSA via its N-terminal lysine, and the conjugate was used to immunize rabbits. To remove non-specific antibodies that might react with the BSA domain of the conjugate, the crude serum was first absorbed with CNBr-activated sepharose 4B resin (Amersham) to which BSA had covalently attached. The peptide-specific antibody was then purified with the resin and used for immunocytochemical localization of VER2 in wheat.

### Immunocytochemistry

Plumules were fixed in 4% formaldehyde and 1% glutaraldehyde in phosphate buffered saline (PBS, 0.1 mol/L Na_2_HPO_4_, NaH_2_PO_4_, pH 7.4) at 4°C for 4 h. Materials were then washed in PBS and dehydrated in ethanol, then 10-µm transverse sections of paraplast (Sigma) were prepared. Prior to incubation with the primary antibody, the sections were blocked with 1% (w/v) BSA in PBS at 37°C for 30 min. Purified peptide antibody was diluted 1∶50 in PBS supplemented with BSA and applied to sections at 37°C for 1 h. The sections were washed 3 times for 10 min each with 0.05% (v/v) Tween 20 in PBS (PBST, pH 7.4) and incubated with the secondary antibody, alkaline phosphatase-conjugated goat anti-rabbit IgG (diluted 1∶500 in PBST), at 37°C for 1 h, then washed in PBST and distilled water. The alkaline phosphatase activity was detected by use of 4-nitro blue tetrazoliu chloride (NBT) and 5-bromo-4-chloro-3-indolyl phosphate (BCIP), according to the manufacturer's instructions (SABC). In control experiments, the primary antibody was replaced with preimmune rabbit serum. Sections were photographed before or after being mounted with 50% glycerol, under bright-field microscopy. The nuclei were indicated by hematoxylin staining.

### SDS-PAGE and western blot analysis

Proteins were separated on 12% SDS-PAGE and electroblotted onto nitrocellulose sheets. Membranes were blocked for 1 h with 5% BSA in TBS-Tween buffer (Tris-HCL 20 mM, NaCL 150 mM, and Tween 0.05%, pH 8.0). Immunoprobing of VER2 was conducted with the anti-VER2 polyclonal antibody for 2 h at 4°C at a dilution of 1∶500 in TBS. Three washes of 5 min each were performed with TBS-Tween. An anti-rabbit IgG conjugated with alkaline phosphatase was used as the secondary antibody at 1∶1000 dilution for 1 h at room temperature. Three washes of 5 min each were performed with TBS-Tween, and target proteins were visualized by incubating the membranes with nitroblue tetrazolium/5-bromo-4-chloro-3-indolyl phosphate (NBT/BCIP). For ECL analysis, the secondary antibody was conjugated with horseradish peroxidase (HRP), and the membrane was exposed to film.

### Transgenic *Arabidopsis*

All Arabidopsis plants used were ecotype C24. Seedlings were grown in a greenhouse under 16 h light/8 h dark. The *VER2* ORF from a full-length *VER2* cDNA was amplified by PCR with the following primers: 5′-CCGTCTAGAATGGCCAAATTCCAGATTAC-3′ (with an XbaI site) and 5′-CCAGGTACCGACCGTGTAAACACCAAATG-3′ (with a KpnI site). The amplified fragment was digested with XbaI and KpnI and cloned into available sites of pBI121-GFP, which contains the *GFP* driven by a cauliflower mosaic virus (CaMV) 35S promoter. *VER2* was located between *35S* and *GFP*. The construct was verified by DNA sequencing.

The pBI121 plasmids carrying *35S::VER2-GFP* and *35S::GFP* were transformed into *Agrobacterium tumefaciens* strain GV3101 separately, and the resulting bacteria were used to transform *Arabidopsis thaliana* ecotype C24. The transformants were selected on plates of Murashige and Skoog (MS) medium containing 50 µg/ml kanamycin. Before being transferred to the greenhouse, transformants were vernalized at 4°C for 3 days. The leaves of transgenic plants were used to observe GFP fluorescence. For some confocal microscopy observations, leaves were counterstained with propidium iodide (PI) (Sigma-Aldrich) to show nuclei.

### Isoelectric focusing and SDS-PAGE

For 2-D electrophoresis, samples and immobiline drystrip gels (7 cm, pH 3–10, Amersham) were rehydrated in a solution containing 9 M urea, 0.5% CHAPS, 0.3% DTT, 0.5% pharmalyte and 0.002% bromophenol blue at 20°C for 12 h. The samples were then run on immobiline gels at 500 V for 0.5 h, then 1000 V for 0.5 h and, finally, 5000 V for 1.5 h. After isoelectrofocusing, gels were denatured first in a balance buffer (50 mM Tris-CL, pH 8.8, 6 M urea, 30% sucrose, 2% SDS, and 0.002% bromophenol blue) containing 1% DTT for 15 min, then transferred to a balance buffer containing 2.5% iodoacetamide for another 15 min. Finally, proteins were resolved by 12% SDS-PAGE.

### Immunoprecipitation

For phosphatase treatment of VER2 protein, extracts of vernalization- and devernalization-treated wheat seedlings were immunoprecipitated by use of anti-VER2 antiserum. Phosphatase treatment followed the manufacturer's instructions (Biolab). The treated proteins underwent western blot analysis to detect the VER2 protein.

For analysis of *O*-GlcNAc modification of VER2 in vernalization- and devernalization-treated wheat seedlings, the monoclonal antibody against *O*-GlcNAc was used to immunoprecipitate *O*-GlcNAc-modified proteins. VER2 in immunoprecipitated proteins was detected by immnoblotting with the polyclonal anti-VER2 antibody.

### Confocal microscopy and propyzamid treatment of *Arabidopsis* leaves

Confocal images were taken under a LSM 510 META confocal laser scanning microscope (ZEISS). Three-week-old *Arabidopsis* cotyledons were used to examine the effect of propyzamid on the distribution of VER2-GFP. The final concentration was 5 µM for propyzamid dissolved in DMSO.

### Mutagenesis

The VER2-S33G and VER2-T209A mutants were generated using PCR method. The two mutant expression constructs of pBI121-VER2-S33G-GFP and pBI121-VER2-T209A-GFP were sequenced to ensure generating the desired mutation.

### Electron microscopy

Leaf fragments (1 to 2 mm) of *Arabidopsis* plants transformed with *35S::VER2-GFP* were fixed in a buffer containing 2% formaldehyde (in 0.1 M PBS, pH 7.4) and 0.5% glutaraldehyde at 4°C for 2 h. They were then rinsed and dehydrated in an ethanol series. Dehydrated materials were embedded in London Resin White (Sigma). Ultrathin sections were prepared and collected on nickel grids precoated with 0.25% formvar. For immunogold labeling, grids were incubated with GFP antibody (diluted 1∶400), then gold-coupled goat anti-rabbit antibody (1∶30). Control sections were treated with preimmune serum. Sections were stained with 2% uranyl and acetate and examined by use of a JEM-1230 transmission microscope (Japan) operating at 80 kV.

## Supporting Information

Figure S1 Lane1, molecular weight markers; The protein markers transferred to nitrocellulose sheet were indicated by staining with ponceau S. Lane2, Western blotting analysis of VER2 in wheat plants vernalized for 3 weeks.
(8.56 MB TIF)

Figure S2 Organ-specific accumulation of VER2 protein during heading stage in winter wheat. (A) Heading stages for analysis. S1, before heading stage; S2, during heading stage. Corresponding panicles were shown below. (B) Expression patterns and abundance of VER2 during different development stages of panicle. Expression level of tubulin detected by immunoblotting was used as loading control.
(2.82 MB TIF)

## References

[1] Peumans WJ, Hause B, Van Damme EJ (2000). The galactose-binding and mannose-binding jacalin-related lectins are located in different sub-cellular compartments.. FEBS Lett.

[2] Van Damme EJ, Lannoo N, Fouquaert E, Peumans WJ (2004). The identification of inducible cytoplasmic/nuclear carbohydrate-binding proteins urges to develop novel concepts about the role of plant lectins.. Glycoconj J.

[3] Zhang W, Peumans WJ, Barre A, Astoul CH, Rovira P (2000). Isolation and characterization of a jacalin-related mannose-binding lectin from salt-stressed rice (Oryza sativa) plants.. Planta.

[4] Grunwald I, Heinig I, Thole HH, Neumann D, Kahmann U (2007). Purification and characterisation of a jacalin-related, coleoptile specific lectin from Hordeum vulgare.. Planta.

[5] Chen Y, Peumans WJ, Hause B, Bras J, Kumar M (2002). Jasmonic acid methyl ester induces the synthesis of a cytoplasmic/nuclear chito-oligosaccharide binding lectin in tobacco leaves.. Faseb J.

[6] Lannoo N, Peumans WJ, Pamel EV, Alvarez R, Xiong TC (2006). Localization and in vitro binding studies suggest that the cytoplasmic/nuclear tobacco lectin can interact in situ with high-mannose and complex N-glycans.. FEBS Lett.

[7] Hart GW, Housley MP, Slawson C (2007). Cycling of O-linked beta-N-acetylglucosamine on nucleocytoplasmic proteins.. Nature.

[8] Wells L, Vosseller K, Hart GW (2001). Glycosylation of nucleocytoplasmic proteins: signal transduction and O-GlcNAc.. Science.

[9] Wells L, Whelan SA, Hart GW (2003). O-GlcNAc: a regulatory post-translational modification.. Biochem Biophys Res Commun.

[10] Zachara NE, Hart GW (2004). O-GlcNAc a sensor of cellular state: the role of nucleocytoplasmic glycosylation in modulating cellular function in response to nutrition and stress.. Biochim Biophys Acta.

[11] Slawson C, Zachara NE, Vosseller K, Cheung WD, Lane MD (2005). Perturbations in O-linked beta-N-acetylglucosamine protein modification cause severe defects in mitotic progression and cytokinesis.. J Biol Chem.

[12] Kneass ZT, Marchase RB (2004). Neutrophils exhibit rapid agonist-induced increases in protein-associated O-GlcNAc.. J Biol Chem.

[13] Gewinner C, Hart G, Zachara N, Cole R, Beisenherz-Huss C (2004). The coactivator of transcription CREB-binding protein interacts preferentially with the glycosylated form of Stat5.. J Biol Chem.

[14] Cole RN, Hart GW (2001). Cytosolic O-glycosylation is abundant in nerve terminals.. J Neurochem.

[15] Comer FI, Hart GW (2000). O-Glycosylation of nuclear and cytosolic proteins. Dynamic interplay between O-GlcNAc and O-phosphate.. J Biol Chem.

[16] Lefebvre T, Cieniewski C, Lemoine J, Guerardel Y, Leroy Y (2001). Identification of N-acetyl-d-glucosamine-specific lectins from rat liver cytosolic and nuclear compartments as heat-shock proteins.. Biochem J.

[17] Thornton TM, Swain SM, Olszewski NE (1999). Gibberellin signal transduction presents ellipsisthe SPY who O-GlcNAc'd me.. Trends Plant Sci.

[18] Jacobsen SE, Binkowski KA, Olszewski NE (1996). SPINDLY, a tetratricopeptide repeat protein involved in gibberellin signal transduction in Arabidopsis.. Proc Natl Acad Sci U S A.

[19] Hartweck LM, Scott CL, Olszewski NE (2002). Two O-linked N-acetylglucosamine transferase genes of Arabidopsis thaliana L. Heynh. have overlapping functions necessary for gamete and seed development.. Genetics.

[20] Hartweck LM, Genger RK, Grey WM, Olszewski NE (2006). SECRET AGENT and SPINDLY have overlapping roles in the development of Arabidopsis thaliana L. Heyn.. J Exp Bot.

[21] Yong WD, Xu YY, Xu WZ, Wang X, Li N (2003). Vernalization-induced flowering in wheat is mediated by a lectin-like gene VER2.. Planta.

[22] Michaels SD, Amasino RM (1999). FLOWERING LOCUS C encodes a novel MADS domain protein that acts as a repressor of flowering.. Plant Cell.

[23] Sheldon CC, Rouse DT, Finnegan EJ, Peacock WJ, Dennis ES (2000). The molecular basis of vernalization: the central role of FLOWERING LOCUS C (FLC).. Proc Natl Acad Sci U S A.

[24] Sung S, Amasino RM (2004). Vernalization and epigenetics: how plants remember winter.. Curr Opin Plant Biol.

[25] Kim SY, He Y, Jacob Y, Noh YS, Michaels S (2005). Establishment of the vernalization-responsive, winter-annual habit in Arabidopsis requires a putative histone H3 methyl transferase.. Plant Cell.

[26] Trevaskis B, Bagnall DJ, Ellis MH, Peacock WJ, Dennis ES (2003). MADS box genes control vernalization-induced flowering in cereals.. Proc Natl Acad Sci U S A.

[27] Trevaskis B, Hemming MN, Dennis ES, Peacock WJ (2007). The molecular basis of vernalization-induced flowering in cereals.. Trends Plant Sci.

[28] Bastow R, Mylne JS, Lister C, Lippman Z, Martienssen RA (2004). Vernalization requires epigenetic silencing of FLC by histone methylation.. Nature.

[29] Sung S, Amasino RM (2004). Vernalization in Arabidopsis thaliana is mediated by the PHD finger protein VIN3.. Nature.

[30] Schmitz RJ, Sung S, Amasino RM (2008). Histone arginine methylation is required for vernalization-induced epigenetic silencing of FLC in winter-annual Arabidopsis thaliana.. Proc Natl Acad Sci U S A.

[31] Wang X, Zhang Y, Ma Q, Zhang Z, Xue Y (2007). SKB1-mediated symmetric dimethylation of histone H4R3 controls flowering time in Arabidopsis.. Embo J.

[32] Xing L, Chong K, Xu Z, Wang R, Tan K (2004). Protein immunocytochemistry localization by using antibody prepared with sunthesized polypeptide.. Progress in Biochemistry and Biophysics.

[33] Ferrigno P, Posas F, Koepp D, Saito H, Silver PA (1998). Regulated nucleo/cytoplasmic exchange of HOG1 MAPK requires the importin beta homologs NMD5 and XPO1.. Embo J.

[34] Comer FI, Vosseller K, Wells L, Accavitti MA, Hart GW (2001). Characterization of a mouse monoclonal antibody specific for O-linked N-acetylglucosamine.. Anal Biochem.

[35] Van Bruaene N, Joss G, Thas O, Van Oostveldt P (2003). Four-dimensional imaging and computer-assisted track analysis of nuclear migration in root hairs of Arabidopsis thaliana.. J Microsc.

[36] Oakley BR, Morris NR (1980). Nuclear movement is beta-tubulin-dependent in Aspergillus nidulans.. Cell.

[37] Van Damme EJ, Hause B, Hu J, Barre A, Rouge P (2002). Two distinct jacalin-related lectins with a different specificity and subcellular location are major vegetative storage proteins in the bark of the black mulberry tree.. Plant Physiol.

[38] Sanderfoot AA, Ahmed SU, Marty-Mazars D, Rapoport I, Kirchhausen T (1998). A putative vacuolar cargo receptor partially colocalizes with AtPEP12p on a prevacuolar compartment in Arabidopsis roots.. Proc Natl Acad Sci U S A.

[39] Foresti O, daSilva LL, Denecke J (2006). Overexpression of the Arabidopsis syntaxin PEP12/SYP21 inhibits transport from the prevacuolar compartment to the lytic vacuole in vivo.. Plant Cell.

[40] Li YB, Rogers SW, Tse YC, Lo SW, Sun SS (2002). BP-80 and homologs are concentrated on post-Golgi, probable lytic prevacuolar compartments.. Plant Cell Physiol.

[41] Drickamer K, Taylor ME (1993). Biology of animal lectins.. Annu Rev Cell Biol.

[42] Jiang JF, Han Y, Xing LJ, Xu YY, Xu ZH (2006). Cloning and expression of a novel cDNA encoding a mannose-specific jacalin-related lectin from Oryza sativa.. Toxicon.

[43] Van Damme EJ, Barre A, Rouge P, Peumans WJ (2004). Cytoplasmic/nuclear plant lectins: a new story.. Trends Plant Sci.

[44] O'Neill EM, Kaffman A, Jolly ER, O'Shea EK (1996). Regulation of PHO4 nuclear localization by the PHO80-PHO85 cyclin-CDK complex.. Science.

[45] Vashisht AA, Pradhan A, Tuteja R, Tuteja N (2005). Cold- and salinity stress-induced bipolar pea DNA helicase 47 is involved in protein synthesis and stimulated by phosphorylation with protein kinase C.. Plant J.

[46] Vosseller K, Sakabe K, Wells L, Hart GW (2002). Diverse regulation of protein function by O-GlcNAc: a nuclear and cytoplasmic carbohydrate post-translational modification.. Curr Opin Chem Biol.

[47] Zachara NE, Hart GW (2006). Cell signaling, the essential role of O-GlcNAc!. Biochim Biophys Acta.

[48] Ruhland W (1965). Handbuch der pflanzenphysiologie encyclopedia of plant physiology.

[49] Zachara NE, O'Donnell N, Cheung WD, Mercer JJ, Marth JD (2004). Dynamic O-GlcNAc modification of nucleocytoplasmic proteins in response to stress. A survival response of mammalian cells.. J Biol Chem.

[50] Ketelaar T, Faivre-Moskalenko C, Esseling JJ, de Ruijter NC, Grierson CS (2002). Positioning of nuclei in Arabidopsis root hairs: an actin-regulated process of tip growth.. Plant Cell.

[51] Kennard JL, Cleary AL (1997). Pre-mitotic nuclear migration in subsidiary mother cells of Tradescantia occurs in G1 of the cell cycle and requires F-actin.. Cell Motil Cytoskeleton.

[52] DeZwaan TM, Ellingson E, Pellman D, Roof DM (1997). Kinesin-related KIP3 of Saccharomyces cerevisiae is required for a distinct step in nuclear migration.. J Cell Biol.

[53] Holzinger A, Lutz-Meindl U (2002). Kinesin-like proteins are involved in postmitotic nuclear migration of the unicellular green alga Micrasterias denticulata.. Cell Biol Int.

[54] Kaminskyj SGW, Yoon KS, Heath IB (1989). Cytoskeletal interactions with post-mitotic migrating nuclei in the oyster mushroom fungus, Pleurotus ostreatus: evidence against a force-generatingrole for astral microtubules.. Journal of Cell Science.

[55] Pearson RA, Luneborg NL, Becker DL, Mobbs P (2005). Gap junctions modulate interkinetic nuclear movement in retinal progenitor cells.. J Neurosci.

[56] Appenzeller C, Andersson H, Kappeler F, Hauri HP (1999). The lectin ERGIC-53 is a cargo transport receptor for glycoproteins.. Nat Cell Biol.

[57] Nyfeler B, Michnick SW, Hauri HP (2005). Capturing protein interactions in the secretory pathway of living cells.. Proc Natl Acad Sci U S A.

[58] Schrag JD, Procopio DO, Cygler M, Thomas DY, Bergeron JJ (2003). Lectin control of protein folding and sorting in the secretory pathway.. Trends Biochem Sci.

[59] Rodriguez-Boulan E, Gonzalez A (1999). Glycans in post-Golgi apical targeting: sorting signals or structural props?. Trends Cell Biol.

[60] Madrid JF, Castells MT, Martinez-Menarguez JA, Aviles M, Hernandez F (1994). Subcellular characterization of glycoproteins in the principal cells of human gallbladder. A lectin cytochemical study.. Histochemistry.

[61] Liu T, Mirschberger C, Chooback L, Arana Q, Dal Sacco Z (2002). Altered expression of the 100 kDa subunit of the Dictyostelium vacuolar proton pump impairs enzyme assembly, endocytic function and cytosolic pH regulation.. J Cell Sci.

[62] Dettmer J, Hong-Hermesdorf A, Stierhof YD, Schumacher K (2006). Vacuolar H+-ATPase activity is required for endocytic and secretory trafficking in Arabidopsis.. Plant Cell.

[63] Matsuoka K, Nakamura K (1991). Propeptide of a precursor to a plant vacuolar protein required for vacuolar targeting.. Proc Natl Acad Sci U S A.

[64] Holwerda BC, Padgett HS, Rogers JC (1992). Proaleurain vacuolar targeting is mediated by short contiguous peptide interactions.. Plant Cell.

[65] Bednarek SY, Wilkins TA, Dombrowski JE, Raikhel NV (1990). A carboxyl-terminal propeptide is necessary for proper sorting of barley lectin to vacuoles of tobacco.. Plant Cell.

[66] Ahmed SU, Rojo E, Kovaleva V, Venkataraman S, Dombrowski JE (2000). The plant vacuolar sorting receptor AtELP is involved in transport of NH(2)-terminal propeptide-containing vacuolar proteins in Arabidopsis thaliana.. J Cell Biol.

[67] Buse MG, Robinson KA, Marshall BA, Hresko RC, Mueckler MM (2002). Enhanced O-GlcNAc protein modification is associated with insulin resistance in GLUT1-overexpressing muscles.. Am J Physiol Endocrinol Metab.

[68] Kneass ZT, Marchase RB (2005). Protein O-GlcNAc modulates motility-associated signaling intermediates in neutrophils.. J Biol Chem.

[69] Van Damme EJ, Barre A, Mazard AM, Verhaert P, Horman A (1999). Characterization and molecular cloning of the lectin from Helianthus tuberosus.. Eur J Biochem.

