# Supplementary figures and images for: Phosphorylation Modification of Wheat Lectin VER2 Is Associated with Vernalization-Induced O-GlcNAc Signaling and Intracellular Motility
Source: PLoS One. 2009 Mar 16;4(3):e4854. doi: 10.1371/journal.pone.0004854 (PMC2654674; doi:10.1371/journal.pone.0004854)

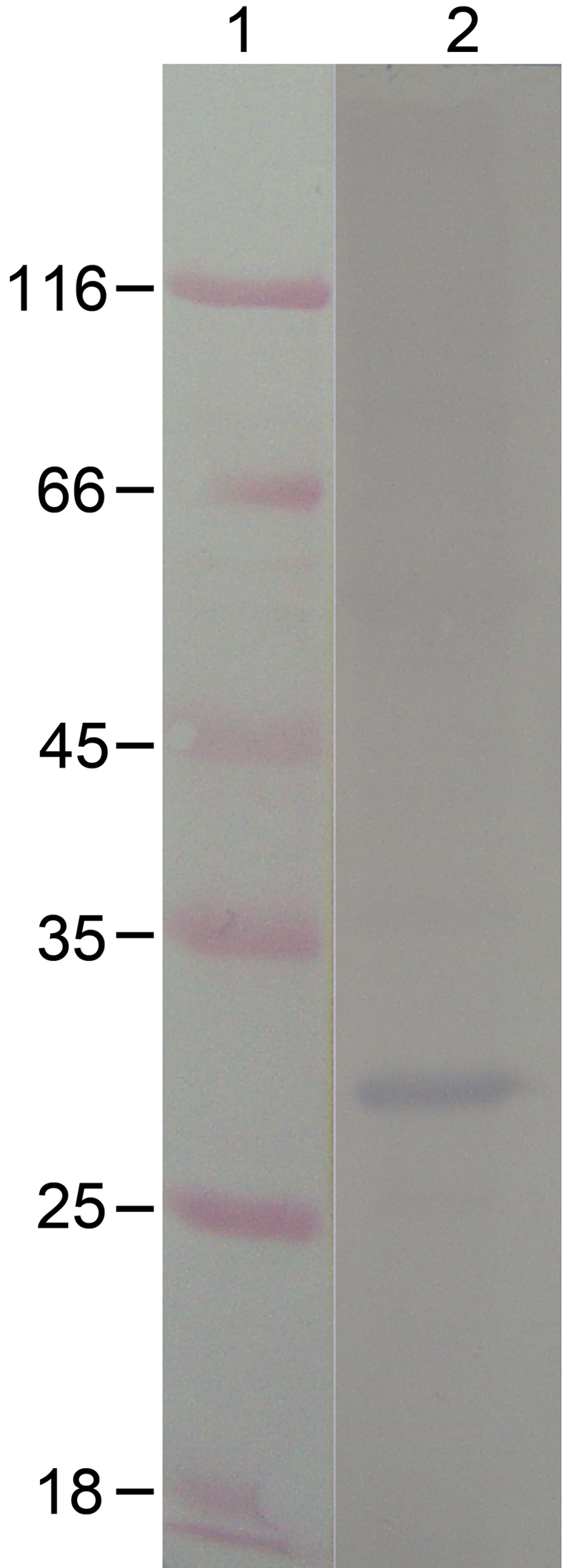

Supplement: Figure S1 — Lane1, molecular weight markers; The protein markers transferred to nitrocellulose sheet were indicated by staining with ponceau S. Lane2, Western blotting analysis of VER2 in wheat plants vernalized for 3 weeks. (8.56 MB TIF) [file pone.0004854.s001.tif]

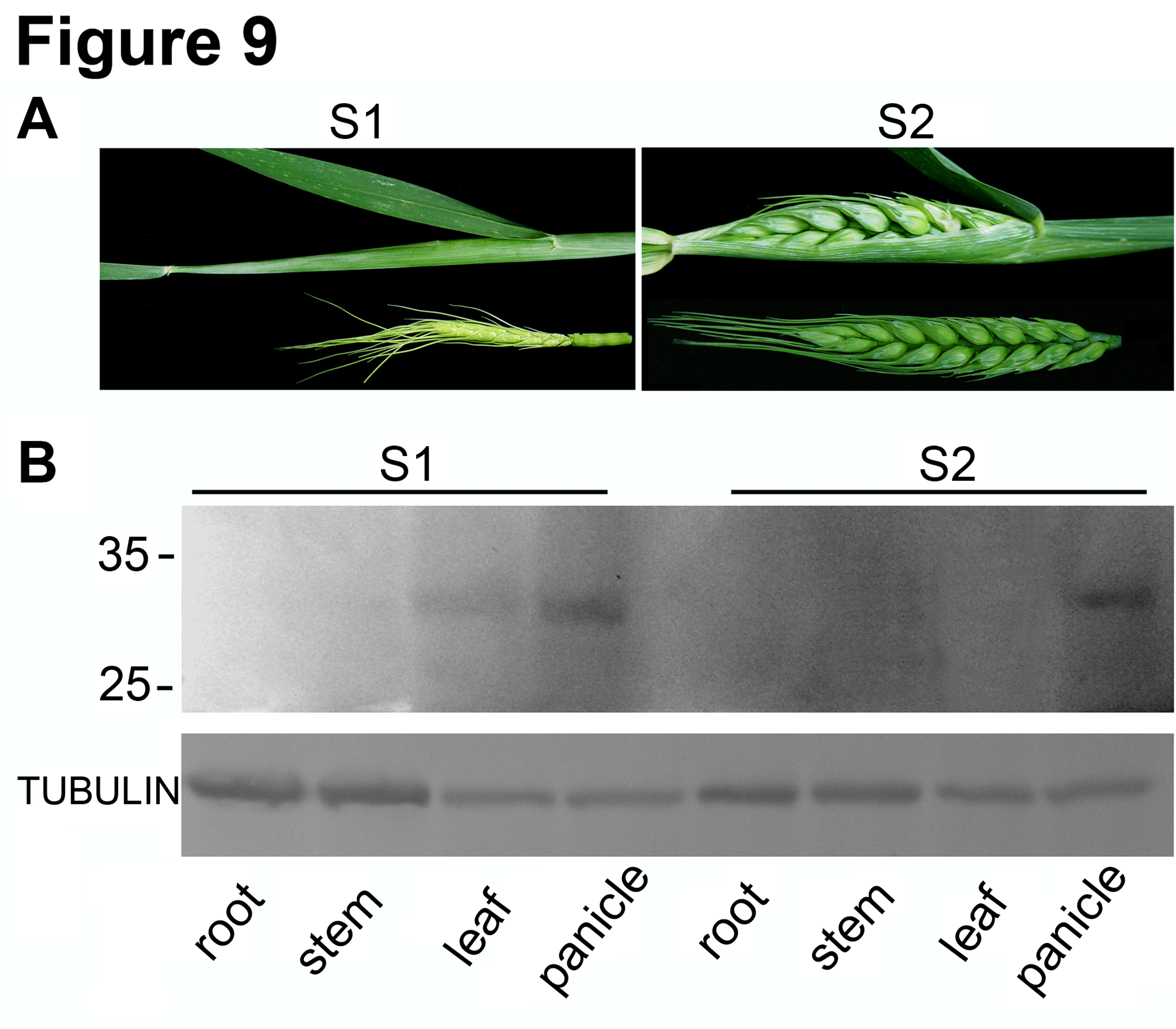

Supplement: Figure S2 — Organ-specific accumulation of VER2 protein during heading stage in winter wheat. (A) Heading stages for analysis. S1, before heading stage; S2, during heading stage. Corresponding panicles were shown below. (B) Expression patterns and abundance of VER2 during different development stages of panicle. Expression level of tubulin detected by immunoblotting was used as loading control. (2.82 MB TIF) [file pone.0004854.s002.tif]
